# Microplastics inhalation and their effects on human health: a systematic review

**DOI:** 10.1093/eurpub/ckac131.152

**Published:** 2022-10-25

**Authors:** G Lombardi, M Di Russo, D Zjalic, T Lanza, M Simmons, U Moscato, W Ricciardi, C Chiara

**Affiliations:** 1 Department of Life Sciences and Public Health, Catholic University of Sacred Heart, Rome, Italy; 2 College of Population Health, Thomas Jefferson University, Philadelphia, USA; 3 Istituto di Planetary Health, Rome, Italy

## Abstract

**Background:**

Microplastics (MPs) are defined as small particles less than 5 mm in size occuring in the environment as a consequence of plastic pollution. MPs are classified into primary MPs, which are created for industrial uses, and secondary MPs, that derive from the degradation of larger plastic items. With the global increase in plastic production, MPs have become widely distributed in the natural ecosystems and have been charged with causing several detrimental effects on both the environment and on human health. Moreover, plastics often include additives to improve their properties, which may produce additional toxic substances. Humans can be exposed to MPs through different pathways, including ingestion, inhalation and dermal contact. The aim of this systematic review is to synthesize whether inhaled microplastics and plastic additives have negative effects on human health.

**Methods:**

MEDLINE, Scopus and Web of Science were searched starting from December 2021. The systematic review was conducted according to the PRISMA guidelines. Eligible studies were primary studies which reported the effects of inhaled MPs on the respiratory system. Appropriate quality assessment tools were used according to the study design of primary studies.

**Results:**

38 studies met the inclusion criteria. Most of the studies were conducted in vitro, while there was a scarcity of papers that investigated the effects of MPs in population cohorts. Preliminary results show that MPs can induce pro-inflammatory or pro-carcinogenic effects by different mechanisms, depending on particles’ concentration, size, type and surface charge.

**Conclusions:**

Literature has underlined several negative health concerns resulting from the absorption of microplastics and plastic additives. By gathering this information, this systematic review sheds light on the possible threats of MPs inhalation to human health and discusses whether an implementation of new public health policies for the foreseeable future is needed.

**Key messages:**

• Inhalation is a major route of exposure to microplastics.

• Inhaled microplastics or plastic additives may have detrimental effects on human health, promoting respiratory diseases or carcinogenic processes.

